# Classic Kaposi's sarcoma in southern Sardinia, Italy

**DOI:** 10.1038/sj.bjc.6602159

**Published:** 2004-09-07

**Authors:** L Atzori, D Fadda, C Ferreli, C Pastorelli, P Iannelli, M Rais, G Faa, P Cocco, N Aste

**Affiliations:** 1 Clinica Dermatologica, Università di Cagliari, Via Ospedale 54, 09124 Cagliari, Italy; 2 Divisione Dermatologia, Ospedale SS. Trinità di Cagliari, Cagliari, Italy; 3 Servizio di Dermatologia, Azienda Ospedaliera Brotzu di Cagliari, Cagliari, Italy; 4 Servizio di Anatomia Patologica, Ospedale Oncologico di Cagliari, Cagliari, Italy; 5 Anatomia Patologica, Università di Cagliari, Cagliari, Italy; 6 Istituto di Medicina del Lavoro, Universita di Cagliari, Cagliari, Italy

**Keywords:** classic Kaposi's sarcoma, epidemiology, southern Sardinia, urban and rural area

## Abstract

The first examination of classical Kaposi's sarcoma incidence in southern Sardinia (Italy) in 1998–2002 found the highest rate recorded in the island of 2.49 per 100 000 per year (standardised).

Kaposi's sarcoma (KS) is a multifocal vascular neoplasm (Ziegler *et al*, 1988), but the nature of the proliferating cells, and whether the lesions represent an exuberant hyperplasia or a true malignancy are still disputed (Buonaguro *et al*, 1995). The association with HHV8 infection in all forms of the disease (Babal and Pect, 2003) is consistent with a common pathologic process, but certain clinical-epidemiological aspects differentiate the course, the prognosis, and treatment of each form. Classical KS corresponds to the original description (Kaposi, 1872), and predominantly affects elderly males, with a higher incidence in southern and eastern European countries, with a predominance among Jews and people of Mediterranean descent (Ziegler *et al*, 1988, Mackie, 1992; Franceschi and Geddes, 1995). Prior to the AIDS epidemic, KS incidence rates were two- to three-fold higher in Italy than in the USA and Sweden, and as much as tenfold higher than in England and Australia (Geddes *et al*, 1994). In north-eastern Sardinia (Italy), classic KS incidence rate is among the highest in the world at 1.59 per 100 000 (Cottoni *et al*, 1996). Sardinia has a Mediterranean homogeneous island population of ancient origin; genetic studies have already demonstrated a positive association of KS with the HLA-DR5 haplotype (Contu *et al*, 1984).

We have investigated whether KS incidence rate is elevated also in the southern part of Sardinia, which includes the capital city Cagliari. We also compared incidence rates in urban and rural areas, as a surrogate for environmental factors in KS aetiology.

## MATERIALS AND METHODS

Incidence of classical Kaposi's sarcoma among the resident population of the Cagliari province, aged ⩾40 years, in 1998–2002 was determined from the registries of all pathology and dermatology departments in southern Sardinia. Only clinically and histologically documented cases of classical KS were considered. Particular care was then taken to avoid including duplicated cases.

### Statistical methods

Standardised incidence of classical KS in 1998–2002 was calculated, together with relevant standard errors both at the province and health district (Local Health Unit) level, using the 2001 Italian census population as the standard. Three local Health Units comprise the subunits of the Cagliari province of which one, comprising Cagliari and its metropolitan area, is mainly urban, the other two being mainly rural. Rates were truncated below the age of 40 years. Person-years for the total province, and for the urban and rural districts, were calculated by 5-year age-groups for the total resident population and by gender. The resident population outside census year (2001) was taken as being the same as in the 2001 census. We calculated the 95% confidence interval of the standardized rates as follows.


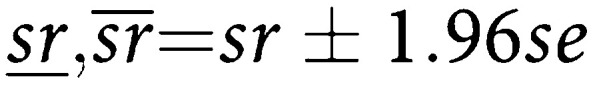


where *sr* and *sr* are respectively the lower and upper bound of the 95% confidence interval of the standardised rate (*sr*), and *se* is its standard error, as derived from the binomial distribution (*se*=√*pq*/*n*, where *p*=rate; *q*=1−*p*; *n*=rate denominator). The analysis was conducted using the SPSS® software.

## RESULTS

Overall, 37 cases of classical KS (22 men and 15 women) were identified in the Cagliari province over the period 1998–2002 (Table 1

**Table 1:** 1998 to 2002 standardised KS incidence rate (× 10^−5^/year) and 95% confidence intervals (CI) in southern Sardinia, by gender and 5-year age-groups

	**Men**		**Women**		**All**	
**Age groups**	***N***	**Rate (95% CI)**	***N***	**Rate (95% CI)**	***N***	**Rate (95% CI)**
40–44	0	0	0	0	0	0
45–49	0	0	0	0	0	0
50–54	1	0.8	0	0	1	0.39
55–59	2	1.86	0	0	2	0.91
60–64	2	2.16	1	0.99	3	1.55
65–69	2	2.47	1	1.07	3	1.72
70–74	5	7.90	5	6.07	10	6.87
75+	10	11.51	8	5.08	18	8.0
All ages ⩾40	22	3.07 (1.91, 4.23)	15	1.96 (1.07, 2.85)	37	2.49 (1.75, 3.22)

The 2001 census Italian population aged ⩾40 years was the standard.

), a standardised incidence rate of 2. 49 × 10^−5^/year (95% C.I. 1.75, 3.22). Among men, the rate was about 1.5-fold of that among women (men: 3.07 per 10^−5^, 95% CI 1.91, 4.23; women: 1.96, 95% CI 1.07, 2.85), and was highest among men aged 75 years or older and among women, in the 70–74-year age group. Average age at diagnosis was 74.6 years (s.d. 10.3), and it was significantly lower among men (men: 72.5 years, s.d. 10.7; women: 77.7 years, s.d. 9.1; *t*=2.97; *P*<0.01). Rates were significantly greater in the urban health district (*sr*=3.46, 95% CI 2.53–4.57) compared to the rural health districts (*sr*=1.14, 95% CI 0.34–1.94), and this was so in both genders.

## DISCUSSION

This is the first study of classic KS incidence in southern Sardinia. The high incidence rates reported in the northern areas (1.59 per 100 000 inhabitants; 2.43 for men; 0.77 for women) (Cottoni *et al*, 1996) are even higher in our study (2.43 per 100 000 inhabitants (2.92 for men; 1.96 for women). We also noted incidence was higher in urban than in rural districts, which is in contrast with previous reports. In the northern area of Sardinia, most cases have been observed in countryside areas, suggesting a role of contact with animals and farming cereals (Cottoni *et al*, 1997). Environmental factors, such as volcanic soil exposure (Montella *et al*, 1997), bloodsucking insects (Ascoli *et al*, 2003), and past history of malaria (Cottoni *et al*, 1997) have been investigated, but with inconsistent findings. Our results, on the other hand, suggest a parallelism with epidemic African and AIDS-related cases (Ziegler *et al*, 1997), in which a higher socioeconomic status has been considered as a marker for either enhanced exposure to sexually transmitted agents, including HHV8, or delayed expression of a childhood infection.

As with other oncogenic viruses, HHV8 infection alone is not sufficient for KS development (Iscovich *et al*, 2000) and further analytical studies are warranted to investigate the role of additional contributory factors.
